# Cocaine enhances HIV-1 gp120-induced lymphatic endothelial dysfunction in the lung

**DOI:** 10.14814/phy2.12482

**Published:** 2015-08-26

**Authors:** Xuefeng Zhang, Susan Jiang, Jinlong Yu, Paula M Kuzontkoski, Jerome E Groopman

**Affiliations:** 1 Division of Experimental Medicine, Beth Israel Deaconess Medical Center, Harvard Medical SchoolBoston, Massachusetts, USA; 2 Department of Psychiatry, Mclean Hospital, Harvard Medical SchoolBelmont, Massachusetts, USA; 3 DynaMed, EBSCO Information ServicesIpswich, Massachusetts, USA

**Keywords:** Cocaine, D4 receptor, fibronectin, HIV, lymphatic endothelial cells, sigma-1 receptor

## Abstract

Pulmonary complications are common in both AIDS patients and cocaine users. We addressed the cellular and molecular mechanisms by which HIV and cocaine may partner to induce their deleterious effects. Using primary lung lymphatic endothelial cells (L-LECs), we examined how cocaine and HIV-1 gp120, alone and together, modulate signaling and functional properties of L-LECs. We found that brief cocaine exposure activated paxillin and induced cytoskeletal rearrangement, while sustained exposure increased fibronectin (FN) expression, decreased Robo4 expression, and enhanced the permeability of L-LEC monolayers. Moreover, incubating L-LECs with both cocaine and HIV-1 gp120 exacerbated hyperpermeability, significantly enhanced apoptosis, and further impaired in vitro wound healing as compared with cocaine alone. Our studies also suggested that the sigma-1 receptor (Sigma-1R) and the dopamine-4 receptor (D4R) are involved in cocaine-induced pathology in L-LECs. Seeking clinical correlation, we found that FN levels in sera and lung tissue of HIV^+^ donors were significantly elevated as compared to HIV^−^ donors. Our in vitro data demonstrate that cocaine and HIV-1 gp120 induce dysfunction and damage of lung lymphatics, and suggest that cocaine use may exacerbate pulmonary edema and fibrosis associated with HIV infection. Continued exploration of the interplay between cocaine and HIV should assist the design of therapeutics to ameliorate HIV-induced pulmonary disorders within the drug using population.

## Introduction

Both cocaine use and HIV infection can damage the lungs, resulting in pulmonary pathology. The etiology of HIV- and cocaine-associated pulmonary dysfunction is likely multifactorial (Afessa et al. 1998; Langa et al. 2003; Rubin and Badesch 2005). Chronic inflammation, abnormal accumulation of extracellular matrix (ECM) proteins, and blood vascular impairment have been proposed to play roles (Farber and Loscalzo 2004; Mirrakhimov et al. 2013). While lymphatic vasculature is essential for normal lung function (Kulkarni et al. 2011; Jakus et al. 2014), to date, its role in HIV- and cocaine-induced pulmonary pathobiology has not been studied.

In the central nervous system, cocaine binds to the dopamine transporter and blocks the reuptake of dopamine. Dopamine acts primarily through five distinct dopamine receptors (D1–D5), which are broadly and differentially expressed in various organ systems and tissues (Beaulieu and Gainetdinov 2011). Dopamine receptors are classically associated with the regulation of adenylate cyclase (AC), cAMP, and protein kinase A (PKA) through G protein-mediated signaling in neurons. D1-class dopamine receptors (D1R and D5R) couple with G proteins and induce AC to generate cAMP and activate PKA. In contrast, when D2-class dopamine receptors (D2R, D3R, and D4R) interact with G proteins, signaling through AC and cAMP is inhibited (Kulkarni et al. 2011). Recent studies have indicated that other receptors, the sigma receptor and the *N*-methyl-d-aspartate receptor, are also involved in cocaine-induced effects (Yao et al. 2011a; Ortinski 2014). In particular, sigma-1R functions as a signal adaptor and amplifier in many cell types (Su and Hayashi 2003), and is believed to play a role in neurodegenerative disorders, drug addiction, and HIV infection and progression (Su and Hayashi 2003; Roth et al. 2005; Yao et al. 2011c). Sigma-1R homozygous mutant mice are fertile and no overt phenotype has been identified so far; however, information on the lymphatic system of these mice has not been investigated and reported (Langa et al. 2003).

The time to greatest effect of cocaine depends on the dose and route of administration of the drug. Injected intravenously, onset of action is within seconds, and peaks within 5 min. When snorted, cocaine is rapidly absorbed through the pulmonary circulation and reaches the central nervous system within seconds. As such, its effects begin within 5 min and typically peak within 30 min. The half-life of cocaine in blood is between 60 and 90 min (Caldwell and Sever 1974; Inaba 1989; Cone et al. 1995).

Pulmonary edema is often found in individuals autopsied for cocaine-related death, and among those that test positive for the drug at autopsy; many had interstitial pneumonitis or fibrosis (Murray et al. 1989; Bailey et al. 1994; Drent et al. 2012). Moreover, interstitial fibrosis associated with cocaine may progress even after drug use stops, resulting in respiratory insufficiency and significant mortality (Pare et al. 1989; O’Donnell et al. 1991). Both in vitro and in vivo studies have demonstrated that cocaine causes rapid vessel constriction by inducing the release of endothelin-1 by ECs, and by inhibiting the expression of vasodilators like nitric oxide (Hendricks-Munoz et al. 1996; Kaufman et al. 2007; Pradhan et al. 2008). Prolonged exposure to cocaine results in vascular ischemia, inflammation, hyperpermeability, and disruption of the vascular endothelial bed. Indeed, chronic cocaine users show increased numbers of circulating ECs and plasma markers of endothelial damage including C reactive protein (CRP) and endothelin-1 (Meng et al. 2003; Saez et al. 2011). Cocaine can also modulate vascular permeability by inducing the expression of various cytokines, including tumor necrosis factor *α* (TNF-*α*) and platelet-derived growth factor *β* (PDGF-*β*), and inhibiting endothelium-derived prostacyclins (Lee et al. 2001; Yao et al. 2011a). In addition, cocaine interferes with leukocyte adhesion by regulating inflammatory mediators such as IL-8 (Mao et al. 1997) and macrophage inflammatory protein-1 (Nair et al. 2000), and key adhesion molecules including intercellular adhesion molecule 1(ICAM-1), platelet endothelial cell adhesion molecule 1 (PECAM-1), and vascular cell adhesion molecule 1 (VCAM-1) (Fiala et al. 1998; Zhang et al. 1998; Chen et al. 2005).

HIV-induced pulmonary complications are well-documented (Inaba 1989; Hendricks-Munoz et al. 1996; Henderson et al. 2004; Murray et al. 2013; Scourfield et al. 2014), and virus-encoded proteins, including gp120 (Ullrich et al. 2000; Kanmogne et al. 2005), Nef (Marecki et al. 2006; Almodovar et al. 2011), Tat (Park et al. 2001), and Vpu (Henderson et al. 2004) have been implicated in pathogenesis due to their vasoactive effects on ECs. In particular, it is believed that altered protein trafficking in vascular ECs and smooth muscle cells may contribute to the pathogenesis of HIV-related pulmonary arterial hypertension (Sehgal 2013). Clinical and laboratory studies have indicated that cocaine use enhance HIV-induced effects on vascular endothelium (Lederman et al. 2008; Dhillon et al. 2011; Yao et al. 2011a, 2011b; Dalvi et al. 2014); Dhillon et al. recently reported that lung tissue with signs of early pulmonary arteriopathy from HIV^+^ cocaine users expressed elevated levels of PDGF and reduced levels of tight junction proteins (TJPs) as compared with lung tissue of HIV^+^ individuals with no history of drug use (Dhillon et al. 2011). Moreover, they demonstrated in vitro that HIV-1 and cocaine induced reactive oxygen species and activated signaling through the Ras/Raf/Erk1/2 pathway, which may act to disrupt TJPs (Dalvi et al. 2014).

The lymphatic system is a unique vascular network that acts in parallel with the circulatory system to regulate key physiological functions, including fluid balance and the immune response (Tammela and Alitalo 2010; Alitalo 2011). While its critical role in the homeostasis of tissues and organs, including the lungs, has been addressed in the literature (Jakus et al. 2014; McNellis et al. 2014; Yao and McDonald 2014), pulmonary lymphatics have not been studied in the context of cocaine and HIV. In our previous studies, we demonstrated that HIV-1 gp120 can disrupt the permeability and integrity of lung lymphatic endothelial cell monolayers by modulating the expression of fibronectin expression and its interaction with the Slit2/Robo4 pathway (Zhang et al. 2012), and Slit2 can alter L-LEC signaling and function through the VEGF-C/VEGFR-3 pathway (Yu et al. 2014). These prior observations prompted us to investigate how cocaine may partner with HIV to induce pulmonary lymphatic vascular damage and dysfunction. We used primary human L-LECs to explore the in vitro effects of cocaine and HIV-1 gp120, and examined human lung and serum specimens from HIV^+^ and HIV^−^ individuals to correlate findings in vivo. Clarifying novel molecular mechanisms by which cocaine and HIV contribute to lung damage and pathophysiology may help design targeted therapeutics to prevent or ameliorate HIV-associated pulmonary disorders in cocaine users.

## Materials and Methods

### Cells

Primary human L-LECs and primary dermal human microvascular endothelial cells (d-HMVECs) were purchased from Lonza, Inc. (Allendale, NJ) and maintained in EBM-2 medium with EGM-2MV SingleQuots (Lonza, Inc.). Cells were cultured in an incubator set to 37°C, 5% CO_2_, and 100% humidity.

### Reagents

Recombinant HIV-1 gp120 protein was obtained through the AIDS Research and Reference Reagent Program, Division of AIDS, NIAID, NIH (Germantown, MD). The experimental control of gp120 was prepared by boiling gp120 for 10 min to inactivate its protein activity while preserving its inherent endotoxin activity. Slit2N was purchased from PreproTech, Inc. (Rocky Hill, NJ). Cocaine hydrochloride (#C5776) was obtained from Sigma-Aldrich Corp. (St. Louis, MO). Double distilled water was the vehicle control used for cocaine hydrochloride. Specific inhibitors for the dopamine-4 receptor (Sonepiprazole hydrate) and the sigma-1 receptor (Metaphit methanesulfonate salt) were purchased from Sigma-Aldrich. Anti-phospho-NF*κ*B, anti-phospho-c-Src, anti-CCR5, anti-CXCR4, and anti-albumin antibodies were purchased from Cell Signaling Technology, Inc. (Beverly, MA). Anti-phospho-paxillin, anti-D2-40, and anti-Robo4 antibodies were purchased from Abcam Inc. (Cambridge, MA). VEGF-C was purchased from R&D Systems, Inc. (Minneapolis, MN). All other antibodies were purchased from Santa Cruz Biotechnology, Inc. (Santa Cruz, CA).

### Vascular permeability

We measured permeability by quantifying the translocation of FITC-conjugated dextran particles through L-LEC monolayers in transwell chambers (Millipore Corp., Bedford, MA). L-LECs monolayers were serum starved for 1 h, and then incubated with various concentrations of cocaine for times indicated, or pretreated with HIV-1 gp120 or its control for 2 h before incubating with cocaine. Dextran particles were added to the upper chambers and 5 min later, fluorescence in lower chambers was assessed with a standard plate reader (BioTek Instruments, Inc., Vinooski, VT). Percent permeability was calculated as the relative fluorescence of media in lower chambers of cocaine-treated or of HIV-1 gp120 + cocaine-treated cells/fluorescence of media in lower chambers of control-treated cells × 100. For assays exploring the involvement of the D4 and sigma-1 receptors in permeability, L-LECs were serum starved as above, then pretreated with D4R inhibitor, sigma-1R inhibitor, or their vehicle controls (DMSO and PBS, respectively) for 1 h, before incubating the cells with cocaine or vehicle control for 1 h, and proceeding as described earlier.

### In vitro wound repair assay

L-LECs were seeded in 24-well plates, grown to confluency, and “wounded” with a pipette tip drawn across the center of the monolayers. After washing three times with 1 × PBS and serum-free media to remove detached cells, EBM-2 media (1% BSA) was added alone (Ctrl), or with HIV-1 gp120, cocaine, HIV-1-gp120 + cocaine, or VEGF-C. Images were captured just after wounding (0 h) and 48 h later, and wound area was measured using ImageJ software (NIH, Bethesda, MD). Percent residual wound area = (wound area after 48 h/wound area at 0 h) × 100.

### Human sera and tissue specimens

All lung specimens were obtained from the National Disease Research Interchange (NDRI) (NIH, Philadelphia, PA). Protocols and consent forms were approved by the NDRI in accordance with an assurance filed with and approved by the U.S. Department of Health and Human Services. The serum samples from normal donors were purchased from Sigma-Aldrich Corp. and the samples from HIV^+^ patients were provided by NDRI. The serum samples were heat inactivated and prepared as described previously (Tjotta et al. 1991) before western blot analysis.

### Immunostaining

Lung tissue sections were prepared by the Pathology Core of Harvard Medical School (Boston, MA) and used for immunohistochemical staining as follows: (1) Deparaffinization was done in xylene (10 min, three times), followed by 100% ethanol, 95% ethanol, 80% ethanol, and 50% ethanol (5 min, two times each). Tissue sections were then rinsed with water and 1 × PBS buffer; (2) Antigen retrieval was performed by boiling in 1 × antigen unmasking solution (Vector Laboratories, Inc., Burlingame, CA) for 30 min and allowed to cool to room temperature. Tissue sections were then washed with water and 1 × PBS; (3) Permeabilization was done by incubating slides in 0.1% Triton X-100 in 0.1% sodium citrate for 30 min on ice. The tissue sections were then stained with specific antibodies using the R.T.U. Vectastain® Universal Kit followed by the DAB Peroxidase (HRP) Substrate Kit, per the manufacturer’s instructions (Vector Laboratories, Inc.).

### Confocal microscopy

Cells were cultured in eight-well chamber slides (Thermo Fisher Scientific, Inc., Carlsbad, CA) and serum starved for 2 h, before treated with indicated agents. Subsequently, cells were fixed with 4% (v/v) paraformaldehyde for at least 1 h at room temperature and permeabilized for 2 min on ice. Cells were then incubated with primary antibodies or their isotype controls overnight at 4°C, and washed three times with 1 × PBS. Fluorescence-conjugated secondary antibodies were added for 30 min at 4°C and the cells were washed three times in 1 × PBS. Finally, the chambers were removed and coverslips were affixed with mounting medium containing DAPI (Vector Laboratories, Inc.). Slides were examined under a Leica TCS-NT laser scanning confocal microscope (Leica Microsystems, Bannockburn, IL).

### TUNEL (terminal deoxynucleotidyl transferase dUTP nick end labeling) assay

L-LECs were incubated in culture media (0.5% BSA) with various concentrations of cocaine alone for 24 h, or pretreated with HIV-1 gp120 for 2 h before incubating with cocaine for 24 h. Cells were then fixed with 4% paraformaldehyde and double-stranded DNA breaks labeled using “In Situ Cell Death Detection Kit, Fluorescein” (Roche Applied Science, Mannheim, Germany) per manufacturer’s protocol before capturing images. Percent apoptosis = No. of apoptotic cells/total no. of cells per randomly chosen frame (40× magnification). Data represent the mean ± SD of three independent experiments.

### Cell stimulation, immunoprecipitation, and western blotting

L-LECs were starved for 2 h in serum-free media, and stimulated as indicated. Cells were then lysed in RIPA buffer (Cell Signaling Technology, Inc.). Immunoprecipitation and western blotting were performed as described previously (Zhang et al. 2012).

### siRNA transfection and assessment

D4R siRNAs and noncoding siRNAs, were purchased from Santa Cruz Biotechnology, Inc. L-LECs were grown to 80% confluence in tissue culture dishes and transfected with specific siRNAs or control siRNAs using HiPerFect transfection reagent from Qiagen, Inc. (Valencia, CA). The knockdown efficiency was assessed by western blot analysis, 48 h later.

### Data analysis

Each experiment was repeated at least three times, and representative blots, images, or graphs are shown in the figures. Student two-tailed, paired, *T*-test or ANOVA were used to determine statistical significance (as indicated). *P <* *0.05 was considered statistically significant.

## Results

### Cocaine modulates the permeability of L-LEC monolayers

**Figure 1:**
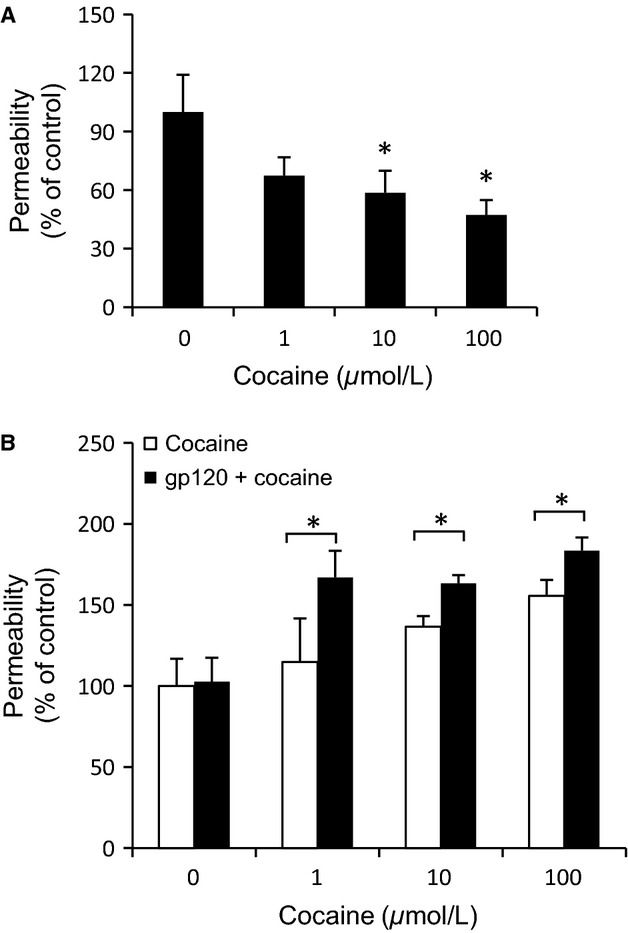
Cocaine modulates the permeability of L-LEC monolayers. (A) Permeability, as determined by the translocation of dextran particles through an L-LEC monolayer. Cells were serum-starved for 1 h and then incubated with cocaine (1, 10, 100 *μ*mol/L) for 2 h before assessing permeability. Data represent the mean ± SD of three independent experiments. *P <* *0.05 by ANOVA (analysis of variance test) for L-LECs incubated with cocaine versus control (0). (B) Cells were serum starved for 1 h and then incubated with cocaine alone (1, 10, 100 *μ*mol/L) for 18 h, or pretreated with HIV-1 gp120 (200 ng/mL) for 2 h before incubating with cocaine, and proceeding as in (A). Data represent the mean ± SD of three independent experiments. *P <* *0.05 by two-way ANOVA for L-LECs incubated with gp120 + cocaine versus cocaine alone.

Cocaine has a potent vasoconstrictive effect on blood vessels (De Giorgi et al. 2012); however, little is known about the effects of cocaine on pulmonary lymphatic endothelium, alone or in the context of HIV-1 infection. To examine this, we evaluated cocaine treatment on the transwell migration of FITC-conjugated dextran beads through primary, L-LEC monolayers. After brief incubation (2 h), we found that cocaine decreased the permeability of L-LECs monolayers in a dose-dependent manner (Fig.1A). Conversely, when L-LECs were incubated with cocaine for a longer time (18 h), the permeability of L-LEC monolayers increased significantly in a dose-dependent manner (Fig.1B, “cocaine”). Such cocaine-induced biphasic effects on lymphatic endothelium are consistent with previous reports of vascular spasm induced after a brief exposure to cocaine, and ischemic injury after sustained exposure (Benzaquen et al. 2001). To assess the possible addictive effect of cocaine, we chose a moderate dose of gp120, which alone did not cause significant changes in permeability. We observed that the combination of cocaine and HIV-1 gp120 significantly enhanced the permeability of L-LEC monolayers versus cocaine alone (Fig.1B). Our data indicate that in vitro cocaine may interfere with the permeability of L-LEC monolayers, alone and in combination with HIV-1 gp120.

### Cocaine induces cell death in cultured L-LECs

**Figure 2:**
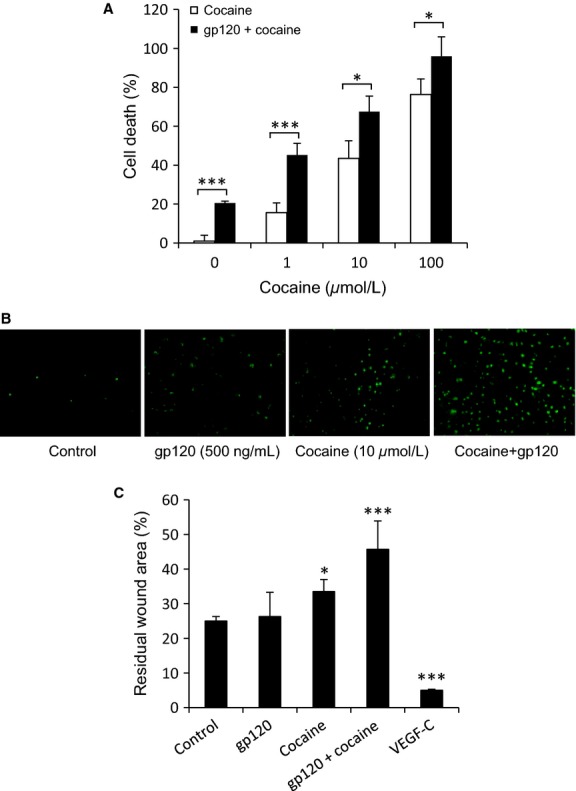
Cocaine-induced toxicity in L-LEC monolayers. (A) Cocaine induces cell death in cultured L-LECs. L-LECs were incubated with cocaine alone (1, 10, 100 *μ*mol/L) for 24 h, or pretreated with HIV-1 gp120 (500 ng/mL) for 2 h before incubating with cocaine and then assessing cell death by TUNEL assay. Data represent the mean ± SD of three independent experiments. *P <* *0.05, ***P <* *0.001 (analysis of variance test) for L-LECs incubated with HIV-1 gp120 + cocaine versus cocaine alone. (B) Representative staining images which were photographed under a fluorescent microscope are shown. (C) Cocaine inhibits in vitro wound healing of L-LECs. L-LEC monolayers were scraped with a pipette tip and then incubated with media alone (Ctrl), with gp120 (500 ng/mL), cocaine (10 *μ*mol/L), gp120 + cocaine, or VEGF-C (50 ng/mL). Monolayers were photographed and measured just after wounding (0 h) and 48 h later. Percent residual wound area = (wound area after 48 h/wound area at 0 h) × 100. Data represent the mean ± SD of three independent experiments. *P <* *0.05, ***P <* *0.001 (Student *T*-test) for control versus all other conditions.

Cocaine can induce apoptosis in blood vascular endothelial cells derived from various tissues (Zhang et al. 1998; He et al. 2000; Dabbouseh and Ardelt 2011). To assess if cocaine has similar effects on pulmonary lymphatic endothelium, we incubated L-LECs with increasing concentrations of cocaine for 24 h, and measured cell death with a TUNEL assay. We also examined the combined effects of cocaine and HIV by incubating these cells with HIV-1 gp120 for 2 h before adding cocaine, as described earlier. We found that cocaine induced dose-dependent cell death in cultured L-LECs (Fig.2A and B), and that the combination of cocaine and HIV-1 gp120 significantly enhanced cell death, as compared with the effects of cocaine alone (Fig.2A and B). These data indicate that cocaine induces cell death in L-LECs and that such an effect can be enhanced by pre-exposure to HIV-1 gp120.

### Cocaine and HIV-1 gp120 inhibit in vitro wound healing of L-LECs

Vascular endothelial cells rapidly replicate to restore endothelial continuity in the hours following vascular vessel damage. Proliferation and migration can be initiated by loss of contact inhibition, cell elongation, and growth factors secreted by endothelial cells themselves and/or by proximal cells (Lancerotto and Orgill 2014). To explore the effects of cocaine and HIV-1 on vessel repair in lymphatic endothelium, we generated artificial wounds by scratching monolayers of L-LECs with a pipette tip, as described previously (Albuquerque et al. 2000). The monolayers were then washed with 1 × PBS and serum-free media, and recultured with media alone (control), or with cocaine and HIV-1 gp120, individually and in combination; VEGF-C served as a positive control. The wounds were photographed and measured at 0 and 48 h (Fig.2C), and percent residual wound area was calculated as described in the Materials and Methods section. Monolayers incubated with VEGF-C showed nearly complete wound repair (Fig.2C). Cocaine alone significantly inhibited in vitro wound repair. The effect of HIV-1 gp120 alone was not statistically significant. However, the combination of cocaine and HIV-1 gp120 significantly impaired wound repair, as compared with cocaine only. These data indicate that cocaine and HIV-1 gp120 may partner to disrupt the healing of damaged pulmonary lymphatic endothelium in vivo.

### Sigma-1R and the D4R are involved in cocaine-induced immediate effects in L-LECs

**Figure 3:**
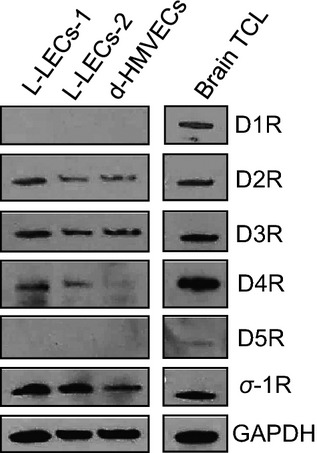
Expression profile of dopamine receptors and sigma-1 receptor (*σ*-1R) in L-LECs. Total cell lysates were harvested with RIPA buffer for western blot analysis. L-LECs-1 and L-LECs-2 = 2 different lots of L-LECs; d-HMEVCs: dermal human microvascular endothelial cells; Human brain total cell lysate (brain TCL) used as positive control. GAPDH used as loading control.

Cocaine can directly or indirectly interact with multiple targets to affect cell signaling and function, including the dopamine receptors, whose expression profiles in endothelial cells vary depending on their tissue of origin (Beaulieu and Gainetdinov 2011). To identify the receptors responsible for the effects of cocaine on lymphatic endothelium, we first examined expression levels of the dopamine receptors (D1R–D5R) and of sigma-1R by western blot analysis in L-LECs, d-HMVECs, and brain total cell lysate (Fig.3). While both types of endothelial cells expressed similar levels of D2 and D3 receptors, L-LECs expressed sigma-1R and D4R at significantly higher levels than d-HMVECs (Fig.3). These data are consistent with a recent report demonstrating the expression of the D2, D3, and D4 receptors in human aortic endothelial cells and umbilical vein endothelial cells by RT-PCR (Ricci et al. 2006). Similarly, we found that D1 and D5 receptor expression was below detection in both L-LECs and d-HMVECs (Fig.3). Based on the prominent role of both sigma-1R and the D4R in endothelial function previously reported and our data, we further explored the contribution of these receptors to cocaine-mediated effects in L-LECs.

**Figure 4:**
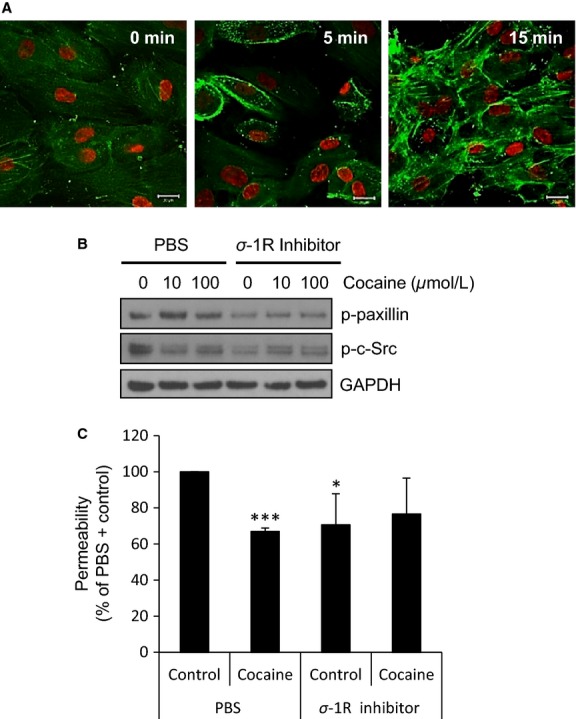
Sigma-1R is involved in cocaine-mediated effects in L-LECs. (A) Cocaine induces phosphorylation of paxillin in L-LECs. L-LECs were serum-starved for 1 h and then incubated with vehicle control (0 min) or 10 *μ*mol/L cocaine for 5 and 15 min. Green = phosphorylated paxillin; red = nuclei. Scale bars = 20 *μ*m. Representative images shown. (B) The effect of sigma-1R inhibition on cocaine-induced phosphorylation of paxillin and c-Src. L-LECs were serum-starved for 1 h, then pretreated with sigma-1R inhibitor (1 *μ*M) or 1 × PBS for 1 h before incubating with cocaine (10 and 100 *μ*mol/L) for 15 min. GAPDH used as loading control. (C) Permeability, as determined by the translocation of dextran particles through an L-LEC monolayer. Cells were serum starved for 1 h and pretreated with sigma-1R (*σ*-1R) inhibitor (1 *μ*mol/L) or 1 × PBS for 1 h before incubating with cocaine (10 *μ*mol/L) or Ctrl for 1 h before assessing permeability. Data represent the mean ± SD of three independent experiments. *P <* *0.05; ***P <* *0.001 by Student *T*-test for permeability of monolayers treated with (PBS + Control) versus all other conditions.

To that end, we evaluated the effects of a specific sigma-1R inhibitor on cocaine-modulated signaling in L-LECs. Because cocaine can alter the cytoskeletal system in endothelial cells and smooth muscle cells (Fiala et al. 2005; Dhillon et al. 2008), and paxillin activation is known to be involved in the cytoskeletal rearrangement of lymphatic endothelial cells (Zhang et al. 2007; Jones et al. 2009), we assessed the effect of cocaine on paxillin activation by examining levels of phosphorylated paxillin in control-treated L-LECs (0 min), and in those incubated with cocaine for 5 and 15 min by confocal microscopy (Fig.4A). We found that cocaine induced a dramatic, time-dependent increase in paxillin activation as compared with L-LECs incubated with the vehicle control (Fig.4A). However, when L-LECs were pretreated with the sigma-1R inhibitor, cocaine-mediated activation of paxillin was blocked. Interestingly, cocaine-induced dephosphorylation of c-Src was also partially reversed by the sigma-1R inhibitor (Fig.4B). Since paxillin and c-Src are key regulatory molecules in endothelial cytoskeletal rearrangement and permeability, our data strongly suggested the involvement of sigma-1R in cocaine-mediated effects.

Next, we examined how sigma-1R may regulate cocaine-induced lymphatic hypopermeability. We treated L-LEC monolayers with a sigma-1R inhibitor or solvent control for 1 h, incubated the cells with cocaine or control for 30 min, and assessed permeability as described earlier. Cocaine consistently induced marked hypopermeability in the L-LEC monolayers (Fig.4C). Of note, the sigma-1R inhibitor itself induced only a small decrease in monolayer permeability and did not affect cocaine-induced hypopermeability significantly (Fig.4C).

**Figure 5:**
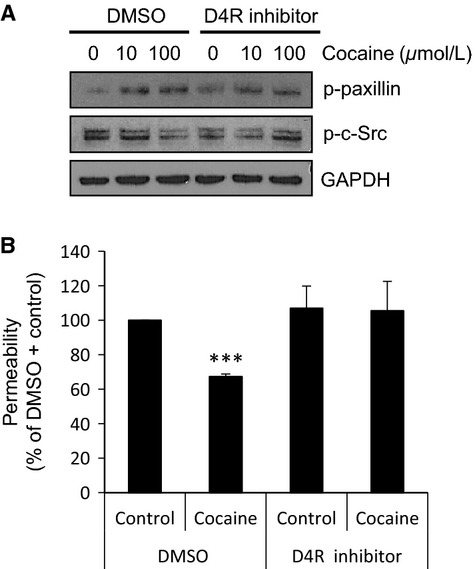
D4R is required in cocaine-mediated effects in L-LECs. (A) The effect of D4R inhibition on cocaine-induced phosphorylation of paxillin and c-Src by western blot analysis. L-LECs were serum-starved for 1 h then pretreated with D4R inhibitor (1 *μ*mol/L) or DMSO for 1 h before incubating with cocaine (10 and 100 *μ*mol/L) for 15 min. GAPDH used as loading control. (B) Permeability was determined as described in Figure4C. Cells were serum-starved for 1 h and pretreated with D4R inhibitor (1 *μ*mol/L) or DMSO for 1 h before incubating with cocaine (10 *μ*mol/L) or Ctrl for 1 h before assessing permeability. Data represent the mean ± SD of three independent experiments. ***P <* *0.001 by Student *T*-test for permeability of monolayers treated with (DMSO + Control) versus all other conditions.

Similarly, we utilized a specific inhibitor of the D4 receptor in order to study its role in cocaine-induced effects in L-LECs. We found that the blocking this receptor reduced cocaine-induced activation of paxillin and significantly blocked cocaine-induced dephosphorylation of c-Src (Fig.5A). When we treated L-LEC monolayers with the D4R inhibitor or solvent control for 1 h, and then incubated the cells with cocaine or control for 30 min, permeability of L-LEC monolayers decreased. This was seen after a brief incubation time with cocaine; however, when the cells were treated with the D4R inhibitor, cocaine-induced hypopermeability was completely reversed (Fig.5B). Taken together, these data suggest that D4R may participate in cocaine-mediated effects on L-LEC permeability through regulating c-Src and paxillin.

### Cocaine enhances the gp120-induced fibronectin expression in L-LECs

**Figure 6:**
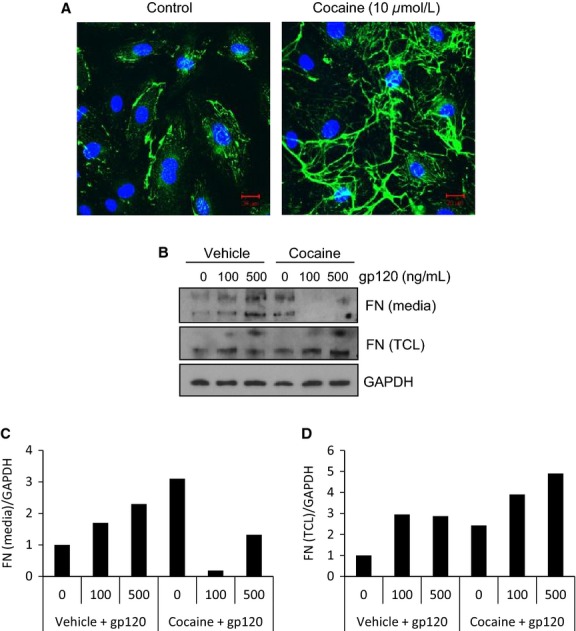
Cocaine induces fibronectin expression in L-LECs. (A) Cocaine-induced FN expression in L-LECs by confocal microscopy. L-LECs were cultured in chamber slides and incubated with cocaine or vehicle control (Ctrl) for 18 h before fixing and staining cells. Green = FN; Blue = DAPI. Scale bars = 20 *μ*m. (B) The effect of cocaine on gp120-induced FN expression in L-LECs and conditioned media by western blot analysis. L-LECs were incubated with cocaine (50 *μ*mol/L) or vehicle control (Ctrl) for 2 h before adding HIV-1 gp120 for another 18 h. FN expression was assessed in the conditioned media and total cell lysates (TCL). GAPDH used as loading control. Densitometry analysis was done using ImageJ software. The results are shown in (C) and (D).

**Figure 7:**
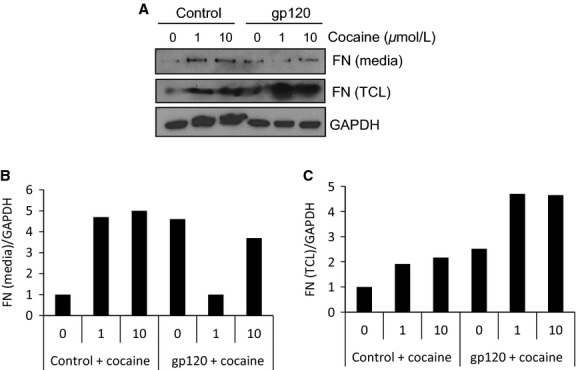
The effect of HIV-1 gp120 on cocaine-induced FN expression in L-LECs and conditioned media. (A) L-LECs were incubated with HIV-1 gp120 (500 ng/ml) or control for 2 h before adding cocaine (0–10 *μ*mol/L) for another 18 h. FN expression was assessed in the conditioned media and total cell lysates. GAPDH used as loading control. The experiment was repeated more than three times. (B and C) Densitometry analysis was done using ImageJ software. The results are shown.

Abnormal accumulation of ECM proteins, including FN, in the interstitial space surrounding endothelial cells contributes to pulmonary vascular dysfunction and fibrosis (Rabinovitch 2001; Tuder et al. 2007). Cocaine can induce the expression of several ECM proteins in human endothelial cells (Gan et al. 1999; Yao et al. 2011c). We and others have demonstrated that HIV-1, as well as HIV-1 envelope protein gp120, induce FN expression in endothelial cells derived from various tissue types, including lymphoid tissues (Torre et al. 2000; Birdsall et al. 2004; Zhang et al. 2012). To characterize how cocaine may modulate FN expression in lymphatic endothelial cells, we measured its effects on FN levels in L-LECs (insoluble form) and in media (soluble form). L-LECs were incubated with increasing concentrations of cocaine for 18 h before harvesting cell lysates. By immunostaining and confocal microscopy, we confirmed cocaine-induced FN expression in cultured L-LECs (Fig.6A). To simulate in vitro, the modulation of FN expression in HIV^+^ cocaine users, we treated L-LECs with cocaine or vehicle control for 2 h before incubating cells with different concentrations of HIV-1 gp120 for an additional 18 h, and measured FN levels in cell lysates and conditioned media by western blot analysis. We found that HIV-1 gp120 significantly increased FN expression in cell lysates in a dose-dependent manner, and that cocaine enhanced this HIV-1 gp120-induced expression (Fig.6B and D*,* “FN [TCL]”). Similarly, we treated L-LECs with HIV-1 gp120 or its control for 2 h before incubating cells with increasing concentrations of cocaine for an additional 18 h, and examined FN expression as above. We again found that cocaine induced FN expression in cell lysates, and that HIV-1 gp120 enhanced this cocaine-induced effect (Fig.7A and C, “FN [TCL]”).

In these experiments, we observed an interesting change in the conditioned media. The levels of soluble FN increased when L-LECs were incubated with either HIV-1 gp120 or cocaine alone; however, when the cells were exposed to both cocaine and gp120, levels of soluble FN decreased significantly (Figs.6, 7, “FN [media]”). Our results indicate that cocaine and gp120, individually, can induce expression of insoluble and soluble FN; however, combined exposure of cocaine and gp120 showed significantly elevated levels of insoluble, cell-associated FN. These data imply that HIV infection plus cocaine use may enhance perivascular or interstitial FN deposition, which can accelerate inflammation and fibrosis in the lungs of HIV^+^ cocaine users.

**Figure 8:**
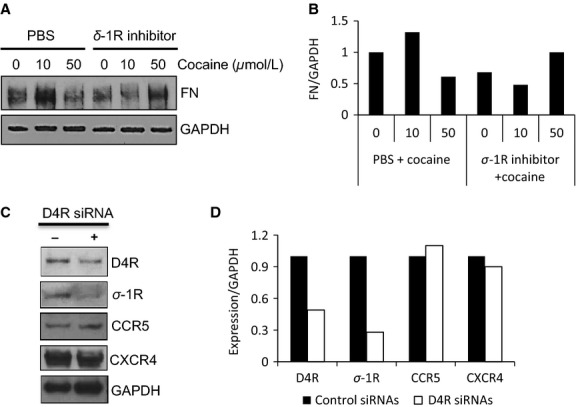
Sigma-1R is involved in cocaine-induced FN expression in L-LECs. (A) The effect of sigma-1R inhibition on cocaine-induced FN expression. L-LECs were serum-starved for 1 h, then pretreated with sigma-1R inhibitor (1 *μ*mol/L) or 1 × PBS for 1 h before incubating with cocaine for 18 h, as indicated. GAPDH used as loading control. The experiment was repeated more than three times. (B) Densitometry analysis was done using ImageJ software. The result is shown. (C) siRNAs to D4R inhibit expression of D4R and of sigma-1R (*σ*-1R) in L-LECs. L-LECs were transfected with D4R-specific siRNAs (+) and with nontargeting siRNAs (−). Protein expression was assessed by western blot analysis 48 h later. GAPDH used as loading control. The experiment was repeated more than three times. The results of densitometry analysis are shown in (D).

In addition, we found that pretreatment with the sigma-1R inhibitor blocked cocaine-induced FN expression in L-LECs (Fig.8A and B). However, the D4R inhibitor had no significant effects on cocaine-induced FN expression (data not shown). Due to the overlapping activities of sigma-1R and the D4R in L-LECs, we performed further experiments to study the potential interaction between D4R and sigma-1R. Using an immunoprecipitation assay, we failed to see a direct association between the two receptors when L-LECs were stimulated with cocaine or its control (data not shown). However, when we transfected L-LECs with D4R-specific siRNAs, both D4R expression and sigma-1R levels were reduced (Fig.8C and D). Since the expression of the HIV-1 coreceptors, CCR5 and CXCR4, and GAPDH expression were not affected (Fig.8C and D), we believe that reduction in sigma-1R was not an off-target effect. Rather, it may be due to a currently uncharacterized association between the D4R and sigma-1R. A previous study has reported that these proteins may bind to the same ligand and interact indirectly (Helmeste et al. 1999).

### Elevated levels of fibronectin in the blood and lungs of HIV-infected patients

**Figure 9:**
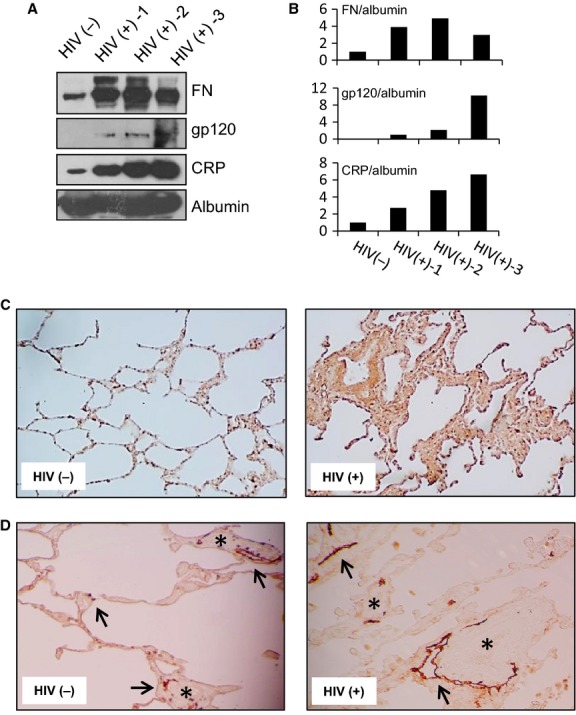
FN levels are elevated in the blood and lung of HIV (+) versus HIV (−) individuals. (A) Representative western blot analysis of FN, HIV-1 gp120, CRP, and albumin expression in heat-inactivated serum samples of HIV (−) and three HIV (+) individuals. Albumin used as loading control. The experiment was repeated three times. (B) Densitometry analysis was done using ImageJ software. The results are shown. (C) FN expression in lung tissue sections (DAB staining, 40× magnification) of HIV (−) and HIV (+) donors. Representative images are shown. (D) Immunostaining with anti-D2-40 antibody (DAB staining, 100× magnification). Representative images are shown. Arrows = lymphatic vessels; asterisks = blood vessels. Immunostaining in (C) and (D) was repeated more than three times.

To pursue our hypothesis that cocaine- and HIV-induced lymphatic damage can contribute to associated pulmonary complications, we measured and compared the levels of FN, HIV gp120, C reactive protein (CRP) with albumin as a control in the serum samples from the HIV (−) and HIV (+) donors by western blot analysis. Significantly higher levels of FN and CRP, biological markers for endothelial injury, were detected in the serum of all three HIV (+) donors versus the HIV (−) donor (Fig.9A and B). HIV-1 gp120 expression confirmed the HIV status of the donors.

We next performed immunostaining to detect FN expression in lung tissue sections. As compared to HIV (−) donors, the lung tissue of HIV (+) donors displayed typical characteristics of chronic inflammation and interstitial fibrosis. Significant accumulation of FN (brown, DAB staining) was seen inside the interstitial spaces of the lung (Fig.9C).

To explore our hypothesis that lymphatic vascular damage and/or dysfunction may contribute to pulmonary complications in the context of HIV and cocaine, we performed immunostaining with anti-D2-40 antibody. As shown in Figure9D, using this specific lymphatic marker, pulmonary lymphatic vessels and capillaries were distinguished from blood vessels and capillaries. The histopathology indicates that lymphatic vessels and capillaries were significantly changed in lung tissues of HIV^+^ individuals, as compared with HIV^−^ individuals (Fig.9D).

### Cocaine modulates Robo4 receptor expression in L-LECs

**Figure 10:**
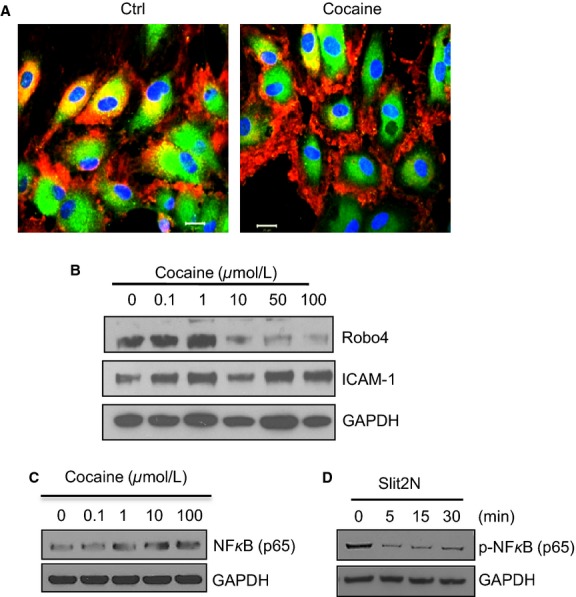
Cocaine modulates Robo4 aggregation and expression in L-LECs. (A) Cocaine-induced Robo4 aggregation in L-LECs by confocal microscopy. Cells were cultured on chamber slides and incubated with control (Ctrl) or cocaine (10 *μ*mol/L) for 30 min before fixing and staining cells. Red = Robo4; Green = eNOS (endothelial nitric oxide synthase); Blue = DAPI. Scale bars = 20 *μ*m. (B) Cocaine inhibits Robo4 expression in L-LECs by western blot analysis. L-LECs were incubated with cocaine (0.1, 1, 10, 50, and 100 *μ*mol/L) for 18 h before assessing protein expression. Expression levels of ICAM-1 were measured after stripping the membrane. GAPDH used as loading control. (C) Cocaine enhances NF*κ*B expression. L-LECs were incubated for 18 h with cocaine (0.1, 1, 10, and 100 *μ*mol/L) before expression levels of NF*κ*B (p65) were assessed by western blot analysis. GAPDH used as loading control. (D) Slit2N inhibits NF*κ*B activation in L-LECs. L-LECs were serum-starved for 2 h, then incubated with Slit2N (1 *μ*g/mL) for 0–30 min before levels of phosphorylated NF*κ*B (p65) were assessed by western blot analysis. GAPDH used as loading control. All experiments were repeated at least three times.

Overexpression of inflammatory cytokines and chemokines by blood endothelial cells has been reported in chronic drug users and those infected with HIV (Araos et al. 2015; de Brito et al. 2015). Recent studies have revealed the anti-inflammatory properties of Slit2 (London et al. 2010; Lim and Lappas 2014) and the stabilizing role of its receptor, Robo4, in endothelium (Jones et al. 2008, 2009). We previously observed that pulmonary endothelial cells express high levels of Robo4 (Zhang et al. 2012; Yu et al. 2014). Pathogens, including HIV and Andes virus, may induce pulmonary vasculopathy by affecting this receptor (Zhang et al. 2012; Gorbunova et al. 2013). Using confocal microscopy, we observed Robo4 aggregation on the cell surface after a brief exposure to cocaine (Fig.10A). Interestingly, cocaine inhibited the expression levels of Robo4 in a dose-dependent manner after longer time exposure (18 h). In contrast, cocaine increased the expression levels of ICAM-1 (Fig.10B). Moreover, we found that cocaine can induce the expression of NF*κ*B, an inflammatory factor, in L-LECs (Fig.10C), and that Slit2N can significantly dephosphorylate NF*κ*B (Fig.10D). These data suggest that sustained exposure to cocaine may contribute to pulmonary lymphatic inflammation, and that manipulation of the Slit2/Robo4 pathway may prevent or ameliorate cocaine- and HIV-induced damage. Further study will address this hypothesis.

## Discussion

Previous studies on cocaine- or HIV-associated pulmonary disorders have focused on respiratory epithelium, blood vascular endothelium, and interstitial spaces (Gingo and Morris 2013; Tseng et al. 2014). In this study, we investigated how cocaine and HIV-1 gp120 may damage lung lymphatic endothelial cells with subsequent lymphatic vascular dysfunction. Our results indicate that cocaine can exacerbate HIV-induced pulmonary lymphatic dysfunction by altering permeability and integrity of this endothelium. Cocaine activation of paxillin and associated cytoskeletal rearrangement of L-LECs may lead to lymphatic vascular contraction and altered permeability. With sustained cocaine exposure, lymphatic vascular endothelium may be permanently damaged.

We also found that both cocaine and HIV-1 gp120 induced the expression of FN in L-LECs, and that L-LECs expressed greater levels of FN after exposure to both cocaine and HIV-1 gp120. Excessive expression and deposition of FN were detected in the blood and the lung tissue of HIV^+^ individuals. More specimens from HIV^+^ individuals and cocaine users are needed to definitely characterize the effects of the drug on pulmonary lymphatic endothelium, alone and in combination with HIV infection.

To the best of our knowledge, this is the first study to address how cocaine may act on pulmonary lymphatic endothelium by interacting with the sigma-1R and the D4R. Sigma-1R, a signal adaptor and amplifier in many cell types (Su and Hayashi 2003), has been reported to be involved in cocaine-enhanced HIV pathology (Roth et al. 2005; Yao et al. 2011c). A recent study reported that amphetamine, a drug that also targets the dopamine system, induced endothelial tissue factor expression in aortic vascular ECs by activating D4R and the MAP kinases, p38 and ERK (Gebhard et al. 2010). Another study reported that dopamine inhibited histamine-induced secretion of von Willebrand factor, a marker of endothelial cell activation, by vascular ECs, via D2R and D4R (Zarei et al. 2006). This research and our data strongly suggest involvement of the sigma-1R and D4R in cocaine-mediated effects in L-LECs, and support a possible coordination between sigma-1R and D4R in mediating activities of cocaine. Because blockade of D4R but not sigma-1R inhibited cocaine effects on the permeability of a monolayer of lymphatic endothelial cells, D4R may be a target molecule which controls cocaine-induced acute effects on endothelium. However, sigma-1R may contribute to cocaine-induced toxicity on lymphatic vascular endothelium after longer periods of exposure due to its biological activities in multiple signaling pathways.

Recent studies have revealed anti-inflammatory properties of Slit2N and the stabilizing role of its receptor, Robo4, in endothelium (Jones et al. 2008, 2009; London et al. 2010). We previously reported that the Slit2/Robo4 signaling pathway plays an important role in HIV-induced endothelial cell pathology (Zhang et al. 2012; Yu et al. 2014). We now report that cocaine also can interfere with this signaling pathway, and regulate key molecules including fibronectin and Robo4. On the basis of our studies and others, we believe that the anti-inflammatory effects of Slit2 may antagonize cocaine-induced acute and/or chronic toxicity on the pulmonary lymphatic network. Further evaluation of how cocaine may act on Slit2/Robo4 signaling in the context of HIV could provide insight into interventions to prevent or ameliorate cocaine- and HIV-induced pulmonary complications.

Taken together, our study provides new insights into how cocaine and HIV-1 gp120 may partner to damage lymphatic endothelium and induce pulmonary complications in HIV^+^ drug users. On the basis of our findings and previous reports, we posit that cocaine may exacerbate HIV-1 gp120-induced pulmonary lymphatic endothelial cell damage in part by modulating sigma-1R and D4R signaling and dysregulating FN expression. Innovative approaches targeting these molecules may be developed in the future to antagonize cocaine-enhanced HIV-1 pathobiology.

## Conflict of Interest

The authors have no associations that might pose conflicts of interest.
